# ***Eucalyptus*** Bark Biochar: Production and Characterization

**DOI:** 10.1021/acsomega.5c10258

**Published:** 2026-02-19

**Authors:** Ariane A. F. Pires, Rafaela S. Resende, João L. Barros, Diego A. Silva, Gabriela T. Nakashima, Gabriela B. Belini, Fabio M. Yamaji

**Affiliations:** † Department of Physics, Chemistry and Mathematics (DFQM), Federal University of São Carlos (UFSCar), Campus of Sorocaba, Rodovia JoãoLeme dos Santos, Km 110, Sorocaba, SP 18052‑780, Brazil; ‡ Department of Environmental Sciences (DCA), Campus of Sorocaba, Federal University of São Carlos (UFSCar), Rodovia João Leme dos Santos, Km 110, Sorocaba, SP 18052-780, Brazil; § Federal Institute of Education Science and Technology of São Paulo, Sorocaba 18043-060, Brazil

## Abstract

In recent years, biochar has garnered increasing attention due to its potential applications in soil amendment, adsorption, and carbon sequestration, which has driven a growing research interest in these areas. Moreover, lignocellulosic biomass is the primary feedstock for biochar production, typically obtained through pyrolysis under limited O_2_ conditions. Within this framework, the present study proposes a sustainable approach that valorizes an environmental byproduct from the forestry sector—*Eucalyptus* bark—for biochar production, aiming to improve soil properties through carbon and mineral supply and pH regulation. The raw material was characterized by determining its proximate composition, as well as its structural and chemical features (XRD and FTIR) and thermal behavior (TGA/DTG). After this initial characterization and the definition of suitable pyrolysis conditions, four treatments were carried out at 300 °C, 350 °C, 400 °C, and 450 °C with a fixed residence time of 2 h. Proximate analysis and yield measurements revealed an inverse relationship between fixed carbon content and gravimetric yield. Furthermore, XRD, FTIR, and TGA analyses confirmed chemical and structural transformations occurring during the thermochemical conversion process. Dynamic and isothermal thermogravimetric tests under an inert atmosphere were conducted to simulate the parameters used in a muffle furnace for larger-scale production. The maximum degradation rate temperature (T_max) of *Eucalyptus* bark was identified as 380 °C, determined from the DTG curve and corroborated by the TG curve. Overall, the biochar produced at 450 °C stood out for its high fixed carbon content, elevated pH, lower volatile matter, and greater thermal stability, which are associated with its amorphous and aromatized structure.

## Graphical abstract

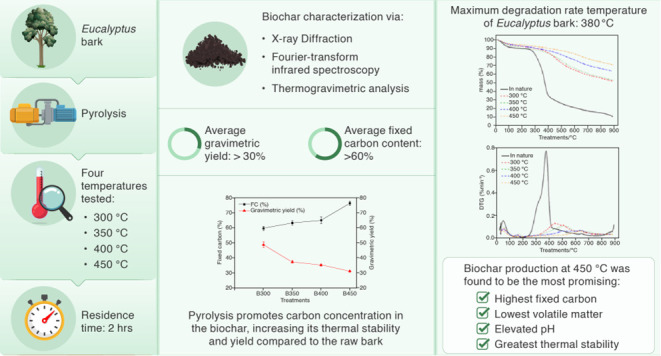

## 1 Introduction

In 2024, of the total 10.2 million hectares of planted trees in Brazil, 7.8 million hectares were *Eucalyptus*, representing 76% of the cultivated area, which highlights its prominent role in the national forestry sector.^1^ As a consequence of this extensive cultivation, large amounts of residues are generated, among which *Eucalyptus* bark stands out. Currently, *Eucalyptus* bark is reused for boiler heating in energy cogeneration systems.^2^ However, this practice has led to several industrial challenges, mainly due to equipment wear caused by inorganic impurities such as sand and mineral salts (calcium, potassium, magnesium, etc.). In addition, these impurities may obstruct the oxidizing air flow in the grates, negatively affecting system efficiency.^3–5^

Thus, there is a clear need to diversify the reuse of this residue, recognizing it as a promising resource for applications that exploit its inherent properties. Particular attention has been given to its chemical composition and to impurities resulting from the harvesting process. In this context, its high ash content, often considered a drawback in energy applications, can be advantageous for alternative uses.^6,7^ One of the possibilities investigated is the use of *Eucalyptus* bark for biochar production. This biochar can subsequently be applied to agricultural soils as a soil conditioner.^8^ Biochar is obtained by heating biomass in an oxygen-limited or oxygen-free system at temperatures above 250 °C. This process, known as carbonization or pyrolysis, is widely used in conventional charcoal production.^9^

At the end of the 19th century, regions of particularly dark and fertile soils were identified in the Amazon. These soils contrasted sharply with the surrounding sandy or clayey soils, which are typically nutrient-poor. Researchers analyzing these areas, known as Amazonian Dark Earths (Terra Preta), determined that they were the product of indigenous activities. These soils are composed of ceramic fragments, bones, and other organic materials, which contribute to their unique properties.^10,11^

Since then, scientific interest in Amazonian Dark Earths has increased due to their high fertility and carbon retention capacity. These characteristics are particularly relevant in the contexto of climate change mitigation, as they contribute to mitigating greenhouse gas emissions. The carbonized matter present in these soils exhibits stability and resistance to decomposition. Such traits align with current research efforts aimed at developing viable strategies to reduce carbon dioxide emissions, the primary greenhouse gas.^10,11^ The World Meteorological Organization (WMO) reported in October 2024 that atmospheric carbon dioxide (CO_2_) concentrations reached a new record in 2023, exceeding 420 ppm. This value represents an increase of approximately 11.4% over the past two decades.^12^

Based on this principle, biochar produced from *Eucalyptus* bark mimics the conditions observed in Amazonian Dark Earths. This approach is consistent with sustainability-driven strategies that prioritize waste valorization and environmental impact reduction. Its application to soil as a final destination aims to improve soil conditions by enhancing fertility with reduced reliance on conventional fertilizers. Additional benefits include pH regulation, carbon supply, and the mitigation of atmospheric carbon emissions, as biochar retains a significant fraction of the biomass carbon.^13,14^

Biochar is a multifunctional material with wide practical relevance. Its applications include soil amendment to improve soil health, nutrient retention, and microbial activity. It is also used for the immobilization of toxic metals and organic pollutants in soil and water systems. In addition, biochar has been explored as a catalyst in industrial processes, for mitigation of greenhouse gas and odor emissions, and as a feed additive to enhance animal health and nutrient absorption.^15^ Biochar also acts as an effective adsorbent capable of removing both organic and inorganic soil contaminants. Simultaneously, it improves soil biochemical properties, enzyme activities, and organic carbon levels, with a key benefit being its strong capacity for nutrient capture and long-term carbon sequestration.^16^

Recent studies highlight biochar’s role as a carbon sink across multiple sectors, including agronomy, livestock production, anaerobic digestion, composting, environmental remediation, construction materials, and energy storage. In these fields, the final reservoirs for biochar are agricultural soils, civil infrastructure, and landfills. Biochar-based fertilizers show promise as nutrient-delivery systems. Similarly, its use as an animal feed has been associated with improved growth performance, balanced gut microbiota, reduced enteric methane emissions, and enhanced productivity. Additionally, biochar improves anaerobic digestion processes by improving biogas yield, removing inhibitory impurities, and supporting process stability. Due to its carbon-rich and stable structure, biochar also promotes plant growth and enables the removal of pollutants, such as heavy metals, organic contaminants, and excess nutrients, from soils and water systems.^17,18^

The objectives of this study were: (i) to produce biochar from *Eucalyptus* bark under four treatments (300 °C, 350 °C, 400 °C, and 450  °C) with a fixed residence time of 2 h; (ii) to characterize the physicochemical properties of both the raw material and the resulting biochar; (iii) to analyze the thermal behavior of the raw material and the products; and (iv) to evaluate the parameters for the optimal pyrolysis condition.

## 2 Materials and Methods

### 2.1 Raw Material

*Eucalyptus* bark was collected in a wood panel industry—Eucatex, located in Salto, São Paulo, Brazil. This residual material from the processing of *Eucalyptus* wood and is derived from hybrid clones of *Eucalyptus grandis* and *Eucalyptus urophylla*. Part of the bark is used in the factory for energy generation, but some of the material is unsuitable for this purpose due to soil impurities remaining on the bark during cultivation and after harvest.

During collection, bark samples were taken from various points within the pile and the company yard, as illustrated in Figure 1. Samples were collected from the base, interior, surfaces, and top of the pile to preserve the characteristics and conditions of the material in its original environment.

**Figure 1:**
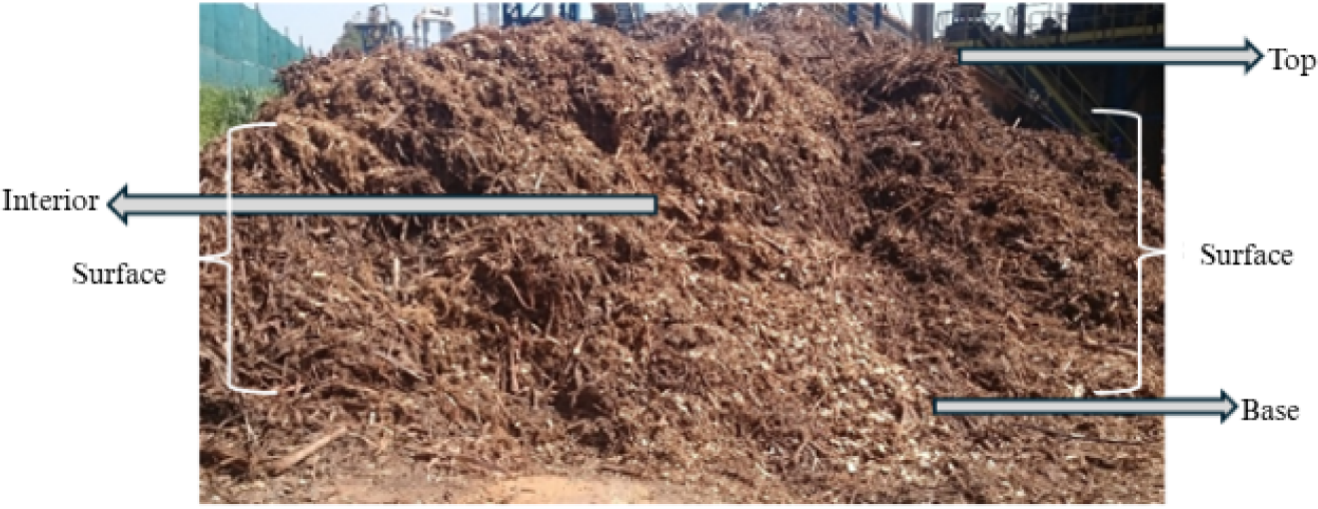
*Eucalyptus* bark in the company yard.

In the laboratory, the bark was first dried in an oven at 103 ± 2 °C for approximately 6 h. It was ground using a Lippel TM-05 grinder equipped with a 4.76 mm screen. The ground material was homogenized following DIN EN 15149-1:2010, as included in BS EN 14780:2011, and the fraction obtained from quaternary sampling was used for physicochemical characterization and biochar production.

### 2.2 Particle Size

Particle size distribution was determined by sieving the ground material through sieves of varying mesh sizes using a Marconi MA 750 orbital shaker with intermittent top tapping at 250 rpm. The bark passed through a three-sieve system with progressive mesh openings: 5 mesh (4.00 mm), 10 mesh (2.00 mm), and 20 mesh (0.841 mm). The percentage of bark retained on each sieve was calculated as eq 1:

1 $$M=\frac{MR}{MT}\times 100$$

where *M* is the percentage retained (%), MT is the total sample mass (g), and MR is the mass retained (g) on each sieve.

### 2.3 Total Extractives

Total extractives (TE) content was determined as the difference between the dry mass of the initial sample and that after sequential extraction, following ASTM D1105-96 (2013) using a cyclohexane-ethanol mixture, ethanol, and hot water. The material was first extracted in a Soxhlet system with cyclohexane-ethanol (1:1, v/v) for 4 h. Dry triplicate samples (∼1 g, 60 mesh) were placed in filter paper envelopes. The second extraction used absolute ethanol in Soxhlet for 4 h, and the final extraction was performed with hot water in a water bath with three cycles, refreshing distilled water after each cycle. After extraction, samples were washed with 500 mL of distilled water and dried at 103 ± 2 °C. Total extractives were calculated as eq 2:

2 $$TE=\left[\frac{Mi-Mf}{Mi}\right]\times 100$$

where TE is the total extractives (%), Mi is the initial dry mass (g), and Mf is the sample mass after extraction (g).

### 2.4 Total Lignin

Total lignin content was determined as the sum of: (i) Klason insoluble lignin (LK), obtained after acid hydrolysis and filtration of the solid residue, and (ii) acid-soluble lignin (LS), quantified in the filtrate by UV–Vis spectrophotometry. The procedures followed the standard methodology for lignocellulosic biomass analysis.

#### 2.4.1 Klason Insoluble Lignin

Insoluble lignin (LK) was measured in triplicate following ASTM D1106-96 (2013). Approximately 1 g of extractive-free, dry sample (60 mesh) was hydrolyzed with 15 mL of 72% H_2_SO_4_ for 2 h under constant stirring, then diluted to 3% acid in a 1000 mL reflux flask with 560 mL of distilled water and refluxed for 4 h. After cooling, the insoluble lignin was filtered, dried at 103 ± 2 °C for 4 h, cooled in a desiccator, and weighed. LK was calculated using eq 3:

3 $$LK=\left(\frac{M1}{M2}\right)\times 100$$

where M1 is the dry mass of Klason lignin (g) and M2 is the initial dry mass of the sample (g).

#### 2.4.2 Soluble Lignin

Soluble lignin (LS) was determined by UV–vis spectrophotometry (200–400 nm) using a Shimadzu UV 3600 spectrophotometer. Absorbances at 215 and 280 nm were measured, with a diluted sulfuric acid solution was used as blank. Soluble lignin concentration (*C*, g/L) was calculated as eq 4:

4 C=[(4.53×A215)−A280]/300

LS (%) was then calculated as eq 5:

5 $$LS=\left(\frac{M1}{M2}\right)\times 100$$

where M1 is the soluble lignin mass (g) and M2 is the initial dry sample mass (g).

### 2.5 Holocellulose

Holocellulose (TH) content was obtained by difference as shows eq 6:

6 TH=100−(TE+LK+LS)

### 2.6 Biochar Production and Gravimetric Yield

Ground bark (5 g per sample) was placed in covered ceramic crucibles to prevent air exposure and heated in a Jung 0212 muffle furnace, in triplicate, at 300 °C (B300), 350 °C (B350), 400 °C (B400), and 450 °C (B450) for 2 h at the target temperature. The choice of temperatures followed the common use of 450 °C for energy-oriented biochar production, while lower temperatures were included to evaluate whether reduced pyrolysis conditions could lower production costs while maintaining acceptable quality. Gravimetric yield (RG) was calculated as eq 7:

7 $$RG=\left(\frac{Mb}{Mc}\right)\times 100$$

where Mb is biochar mass (g) and Mc is initial dry mass (g).

### 2.7 Proximate Analysis

Ash content was determined following ASTM D1102-84 (2013). For each measurement, 1 g of sample was placed in a ceramic crucible and heated in a muffle furnace at 600 °C for 6 h. After cooling completely in a desiccator to room temperature, the crucibles were weighed. Ash content was calculated using eq 8:

8 AC=(M1/M2)×100

where AC is the ash content (%), M1 is the mass of the ash (g), and M2 is the mass of the dry sample (g).

Volatile matter was determined according to ASTM D1762-84 (2007). Approximately 1 g of sample was placed in a covered ceramic crucible and heated in a muffle furnace preheated to 950 °C. The crucible was exposed to open furnace conditions for 3 min, then the furnace was closed and maintained for an additional 6 min. After cooling in a desiccator, the mass of the sample mass was recorded. Volatile matter content was calculated using eq 9:

9 VM=[(M1−M2)/M1]×100

where VM is the volatile matter content (%), M1 is the mass of the dry sample (g), and M2 is the mass after furnace treatment (g).

Fixed carbon content was calculated by difference using the results of ash and volatile matter analyses as shows eq 10:

10 FC=100−(AC+VM)

where FC is the fixed carbon content (%).

### 2.8 pH

Biochar samples (5 g) were mixed with 50 mL distilled water (1:10 ratio), stirred for 1 h at room temperature, allowed to rest for 30 min, and pH measured in triplicate using a GEHACA PG2000 bench pH meter.

### 2.9 Statistical Analysis

Proximate analysis parameters (volatile matter, ash content, and fixed carbon), gravimetric yield, and pH were subjected to analysis of variance (ANOVA) to evaluate differences among the pyrolysis treatments. When significant effects were detected, Tukey’s post hoc test at a 5% significance level was applied. All statistical procedures were performed using RStudio, following standard assumptions of normality and homogeneity of variance.

### 2.10 SEM/EDS

The ashes of *Eucalyptus* bark obtained after muffle furnace combustion were analyzed using Scanning Electron Microscopy (SEM) coupled with Energy Dispersive Spectroscopy (EDS) for structural characterization and semiquantitative elemental analysis. The analysis was performed on a Hitachi TM3000 microscope, at an acceleration voltage of 15 kV.

### 2.11 XRD

Dried, ground, and homogenized samples of raw *Eucalyptus* bark and the produced biochar were analyzed using X-ray Diffraction (XRD). Measurements were carried out on a Shimadzu LABX XRD-6100 diffractometer with a glass sample holder, CuKα radiation (λ = 1.5406 Å), 40 kV voltage, 30 mA current, and scanning range of 2θ: 5° to 65° at a scan rate of 2°/min^–1^.

### 2.12 FTIR

Fourier Transform Infrared Spectroscopy (FTIR) was employed to evaluate chemical functional groups. Milled bark and dried biochar samples were homogenized in a mortar and analyzed using a PerkinElmer Spectrum 65 with Attenuated Total Reflectance (ATR) mode. Spectra were recorded in transmittance mode, with a resolution of 4 cm^–1^, scanning range from 4000 to 600 cm^–1^, and 32 scans per measurement. Sample surfaces were cleaned with 70% ethanol between analyses.

### 2.13 TGA

Thermogravimetric Analysis (TGA) was conducted to assess thermal stability. Samples of 3–5 mg were placed in platinum crucibles under an inert nitrogen atmosphere (N_2_) with a heating rate of 10 °C/min and gas flow of 20 mL/min. The mass was measured using the analytical balance integrated into the TGA equipment, which has a precision of four decimal places. Dynamic TGA analyses ranged from 30 °C to 600 °C. Isothermal analyses maintained each sample at the final treatment temperatures (300, 350, 400, and 450 °C) for 1 h to observe mass loss over time. TG and derivative TG (DTG) curves were plotted using OriginPro 8.5.

## 3 Results and Discussion

### 3.1 Particle Size Distribution

After drying and grinding, *Eucalyptus* bark was sieved to determine particle size distribution and assess the dimensional variation of particles intended for biochar production. Table 1 shows the percentage of mass retained on each sieve used in the procedure.

**Table 1:** Particle Size Distribution of Ground *Eucalyptus* Bark

Sieve (mesh)	Opening (mm)	Retained mass (%)
5	4.00	10.83
10	2.00	52.85
20	0.84	25.68
<20	<0.84	10.65

The ground material retained a relatively coarse particle size compared to finely milled materials. However, using only ground bark (Figure 2) was a deliberate choice to reduce processing steps, saving time and minimizing production costs for commercial-scale biochar.

**Figure 2:**
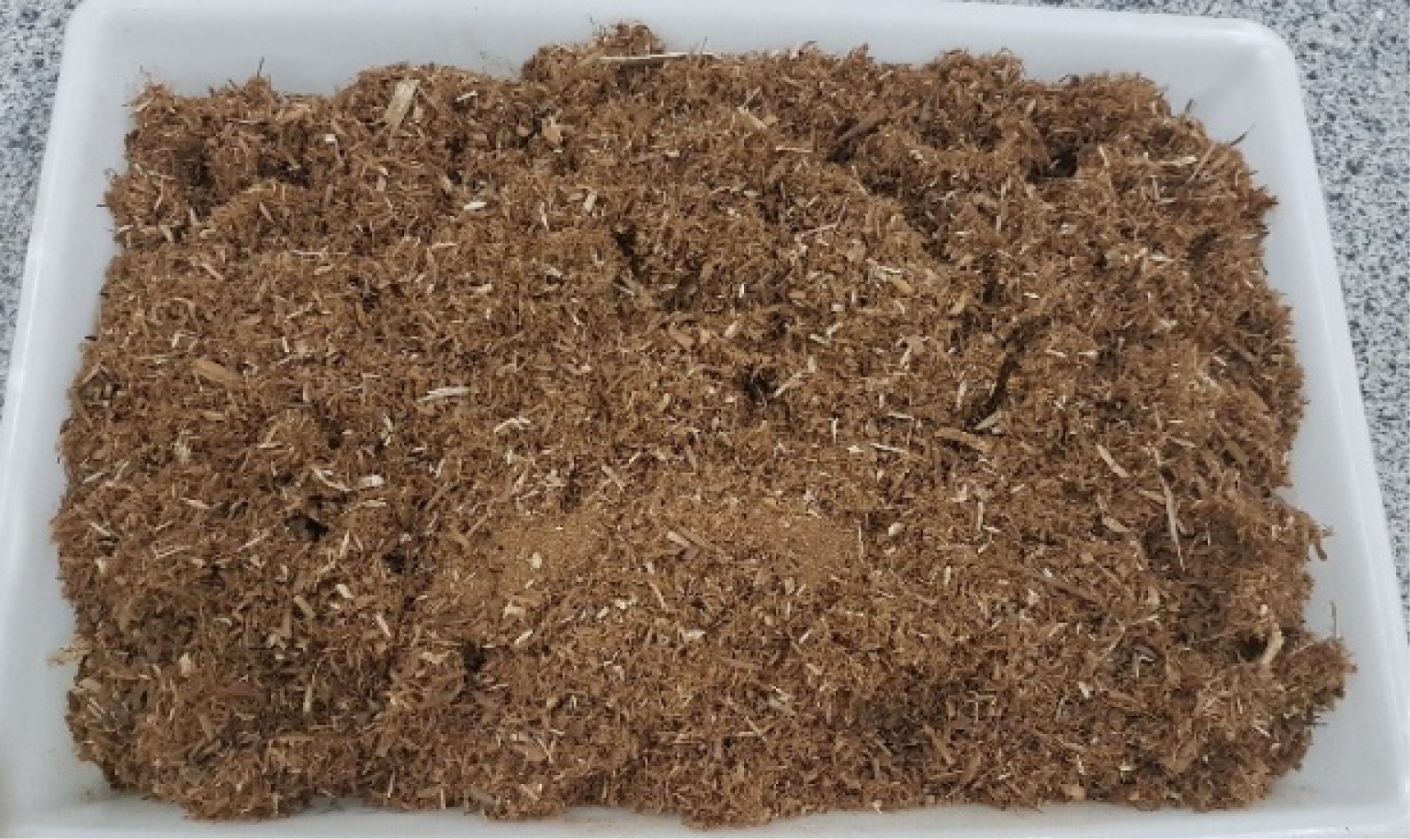
Dried and ground *Eucalyptus* bark used for characterization and biochar production.

### 3.2 Chemical Characterization of *Eucalyptus* Bark

Table 2 presents the results of the major chemical component analysis of *Eucalyptus* bark. Understanding the distribution of primary biomass components is essential because plant material is naturally heterogeneous for smoother readability, influenced by environmental factors such as soil and additives.

**Table 2:** Major Chemical Composition of *Eucalyptus* Bark^a^

Component	Mean Value (%) ± SD
Solvent-extractable compounds	5.14 ± 0.81
Hot water-extractable compounds	12.52 ± 0.79
Total extractives	17.73 ± 1.57
Klason insoluble lignin	15.97 ± 2.13
Soluble lignin	1.68 ± 0.25
Total lignin	17.65 ± 2.35
Holocellulose (*)	64.98 ± 0.84

a (*) Calculated by difference: 100 – (lignin + extractives).

The chemical composition obtained for the *Eucalyptus* bark in this study shows values that are broadly consistent with those reported in the literature, while reflecting the natural variability observed among different species and bark layers. The total extractives measured here (17.73%) are slightly higher than those reported for *E. pellita*—which range from 12.17% to 16.19% depending on the bark fraction^19^—but remain within the general range of 10.30–16.19% documented for other *Eucalyptus* species.^20^

The holocellulose content (64.98%) closely matches the 65.45% reported for mixed bark of *E. pellita*,^19^ and also aligns with values typically reported for bark, where cellulose ranges between 28.7–36.65% and hemicellulose between 18.98–26.2%.^20,21^

The total lignin content found in this study (17.65%) is lower than the values reported for *E. pellita* mixed bark (25.39%)^19^ and those documented for *E. globulus* (28.6–45.37%),^20^ as well as the high lignin proportion cited by Magina et al. (2024) (≈51%).^22^ Such differences are expected due to structural and anatomical variations across species and between inner and outer bark layers; for instance, *E. camaldulensis* shows higher lignin and cellulose contents in the outer bark compared to the inner bark.^21^

In general, the bark exhibited a higher proportion of hot water-soluble extractives, approximately twice that of solvent-soluble extractives. The values for total extractives, total lignin, and holocellulose are consistent with those reported by Teixeira et al. (2016)^49^ for *Eucalyptus* residues, which were 15.88%, 17.12%, and 65.94%, respectively.

The primary biomass components—chemicellulose, cellulose, and lignin—contain the majority of carbon, hydrogen, and oxygen and exhibit distinct thermal behaviors that directly influence the products obtained during pyrolysis.^23,24^ During pyrolysis, these chemical components undergo thermal degradation under controlled temperature and limited oxygen, releasing lower-molecular-weight gases.^25^ Due to its complex structure and thermal resistance, tends to yield more char, whereas cellulose and hemicellulose, being less thermally stable, decompose at lower temperatures, generating larger amounts of bio-oil and gases. Knowledge of these proportions allows optimization of pyrolysis conditions to maximize the desired product—char, bio-oil, or gases.^3,26,27^

### 3.3 Physicochemical Characterization of Biochar

Figure 3 shows images of raw *Eucalyptus* bark (CE) and biochar produced at 300 °C (B300), 350 °C (B350), 400 °C (B400), and 450 °C (B450) with a fixed residence time of 2 h. The progressive darkening of the material with increasing pyrolysis temperature reflects the extent of carbonization achieved at each treatment.^28^

**Figure 3:**
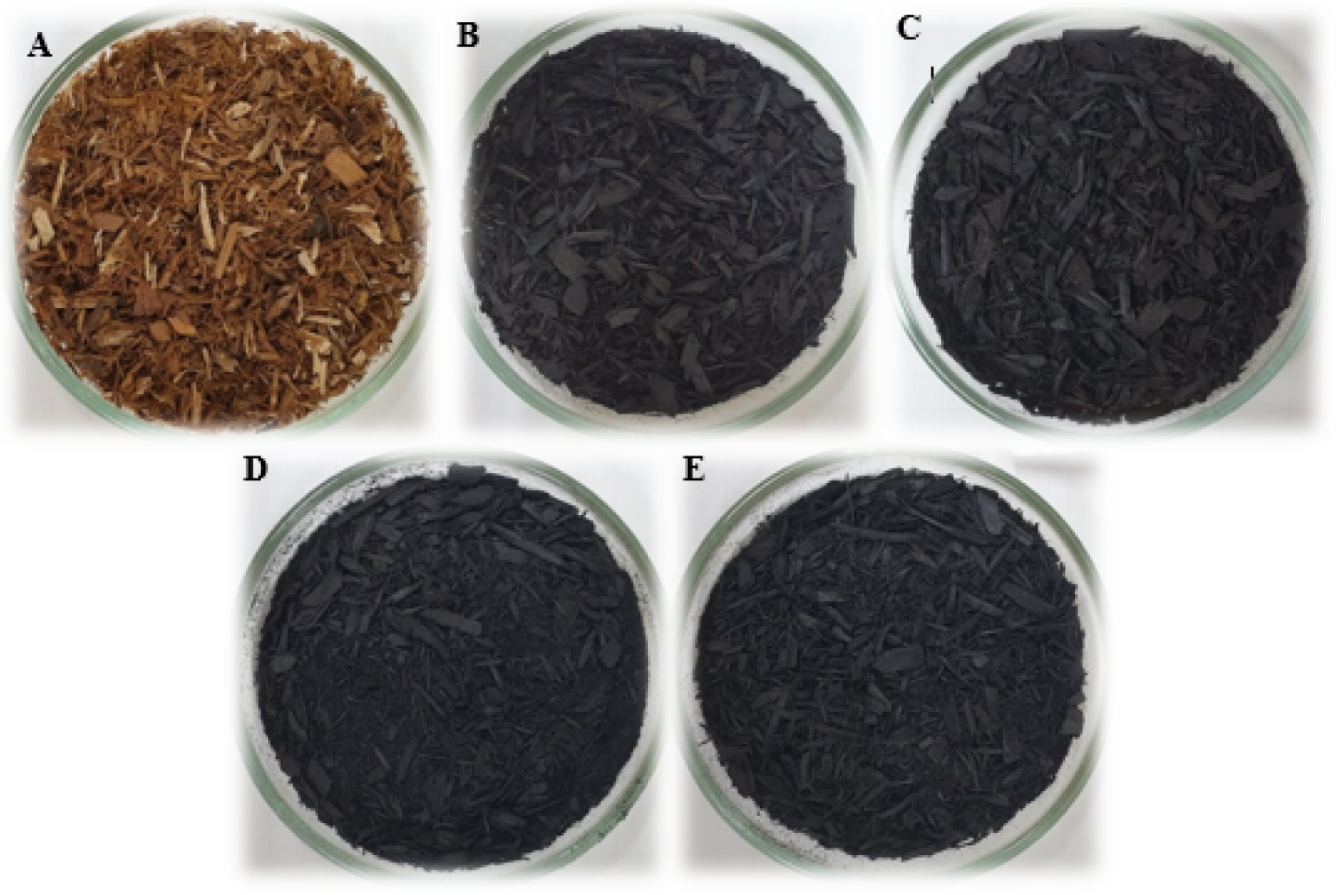
**(A) CE**—raw *Eucalyptus* bark; (B) B300; (C) B350; (D) B400; (E) B450—biochar samples produced at increasing pyrolysis temperatures.

Table 3 presents the proximate analysis of raw bark and all biochar treatments, including gravimetric yield and pH values. Figure 4 shows a comparative graph of proximate analysis and gravimetric yield. Proximate analysis results show statistically significant differences between treatments at different pyrolysis temperatures. Volatile content and pH values varied significantly at 5% significance. Gravimetric yield at 350 and 400 °C did not differ significantly.

**Table 3:** Proximate Analysis, Gravimetric Yield, and pH of Raw Bark and Biochar

Treatment	VM (%)	AC (%)	FC (%)	Gravimetric Yield (%)	pH
CE	73.99 ± 1.24 a	3.49 ± 0.56 a	22.52 ± 1.08 a	–	–
B300	32.42 ± 0.55 b	7.92 ± 0.84 b	59.67 ± 1.37 b	48.85 ± 1.97 a	7.27 ± 0.02 a
B350	27.10 ± 1.65 c	9.55 ± 0.89 bc	63.34 ± 1.56 bc	37.43 ± 0.63 b	7.99 ± 0.02 b
B400	21.99 ± 0.51 d	12.93 ± 1.77 c	65.08 ± 2.27 c	35.43 ± 0.65 b	8.23 ± 0.04 c
B450	12.22 ± 0.35 e	11.58 ± 1.41 bc	76.21 ± 1.44 d	31.28 ± 0.55 c	9.99 ± 0.01 d

**Figure 4:**
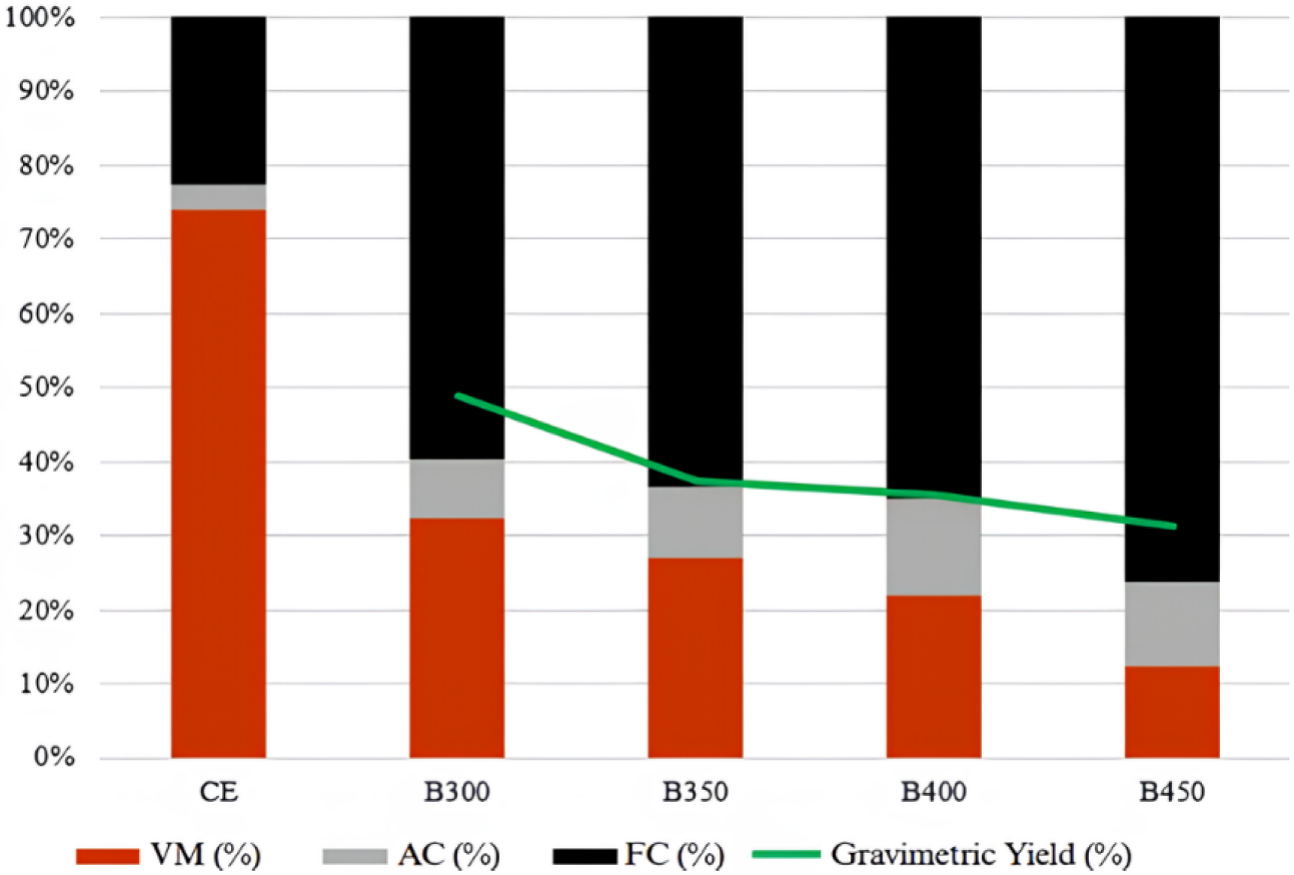
Comparative graph of proximate analysis and gravimetric yield for raw bark (CE) and biochar.

Analyzing Table 3 and Figure 4, it is observed that, compared to the raw bark, the proximate analysis results of the produced biochar differ significantly, particularly showing an increase in fixed carbon and ash content. This was expected, as the thermal treatment promoted the carbonization of *Eucalyptus* bark under limited oxygen conditions, consequently concentrating the carbonaceous and inorganic matter in the resulting biochar.^29^

Values followed by the same letter do not differ significantly at 5% significance according to Tukey’s test.

Figure 4 shows the distribution of components obtained from the proximate analysis, where VM corresponds to volatile matter, AC to ash content, and FC to fixed carbon. The sample labeled CE represents the raw bark, while B300, B350, B400, and B450 correspond to the biochars produced at different pyrolysis temperatures. The green line indicates the gravimetric yield.

As the pyrolysis temperature increases, a progressive decrease in volatile matter and a corresponding increase in fixed carbon can be observed, reflecting a higher degree of carbonization. Ash content also tends to increase due to the concentration of inorganic materials after the thermal degradation of organic components. The gravimetric yield decreases with increasing temperature, as expected, since higher temperatures promote greater mass loss, resulting in biochars with lower volatile content and higher fixed carbon.

The volatile content in the biochar exhibited an inversely proportional behavior to fixed carbon, decreasing as the pyrolysis temperature increased, since these volatiles correspond to lower-molecular-weight gaseous products including carbon monoxide (CO), carbon dioxide (CO_2_), methane (CH_4_), and water (H_2_O).^30^

Ashes, primarily composed of metal oxides, exhibited a considerable increase in relative percentage with rising pyrolysis temperatures. However, this increase in ash content does not indicate an actual change in its absolute quantity within the material but rather reflects a higher relative contribution of ashes to the total mass of the produced biochar. Notably, higher pyrolysis temperatures also corresponded to lower gravimetric yields of biochar.^30,31^

The ability of biochar to neutralize soil acidity has been reported in several studies, highlighting its potential application for pH correction in acidic soils.^32^ As shown in Table 3, the pH values tend to increase with higher pyrolysis temperatures, as organic mass loss rises, O/C and H/C ratios decrease, and inorganic content, i.e., ashes, becomes more concentrated, contributing to the pH outcome.^30^ All measured pH values differed statistically, with B450 exhibiting the highest value, approximately 10. This finding requires practical testing in future experiments, either in the field or at laboratory scale, to clarify its effective influence on soil pH regulation.

Figure 5 presents the relationship between fixed carbon content and gravimetric yield for each treatment, i.e., 300 °C (B300), 350 °C (B350), 400 °C (B400), and 450 °C (B450). Fixed carbon content increased with higher pyrolysis temperatures, while gravimetric yield decreased, indicating an inverse relationship between these variables. Progressive mass loss is typical during biomass pyrolysis, as elevated temperatures promote the degradation of volatile compounds, reducing yield but enriching the fixed carbon fraction. Higher fixed carbon content is particularly relevant for biochar production, as it is directly associated with improved material stability and functionality.

**Figure 5:**
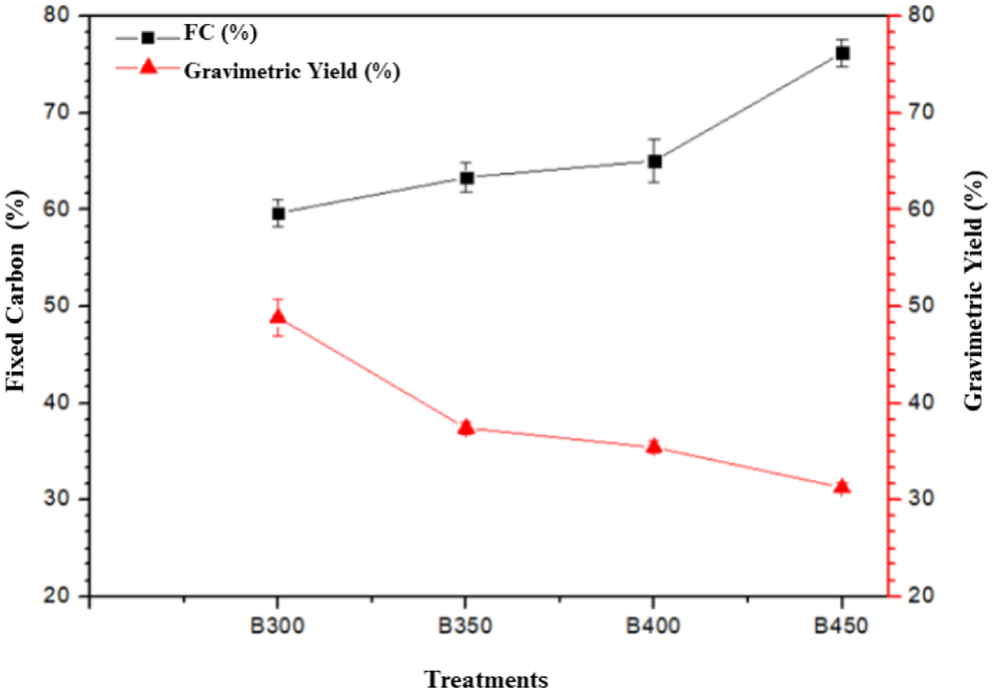
Fixed carbon content and gravimetric yield for the conducted treatments.

Based on the analysis of Table 3 and Figures 4 and 5, the B450 treatment stood out among the others, primarily due to its high fixed carbon content (>75%) and low volatile matter (12%). The elevated fixed carbon reflects a chemically stable composition, resistant not only to temperature but also to environmental degradation, enhancing its potential as a long-term carbon source when applied to soils.

Conversely, the lower volatile matter, approximately half that observed in B350 and B400, is advantageous for soil applications, as it suggests a reduced presence of low-molecular-weight compounds that were eliminated during pyrolysis. These simple-structured compounds are more susceptible to decomposition and can be released as greenhouse gases, such as CO_2_ and CH_4_.

### 3.4 SEM/EDS

Ashes obtained from the complete combustion of *Eucalyptus* bark were analyzed using scanning electron microscopy (SEM). Figure 6A shows the SEM micrograph of the bark ashes at 800× magnification, while Figure 6B presents a higher magnification image (1200×), corresponding to the region analyzed by energy-dispersive X-ray spectroscopy (EDS).

**Figure 6:**
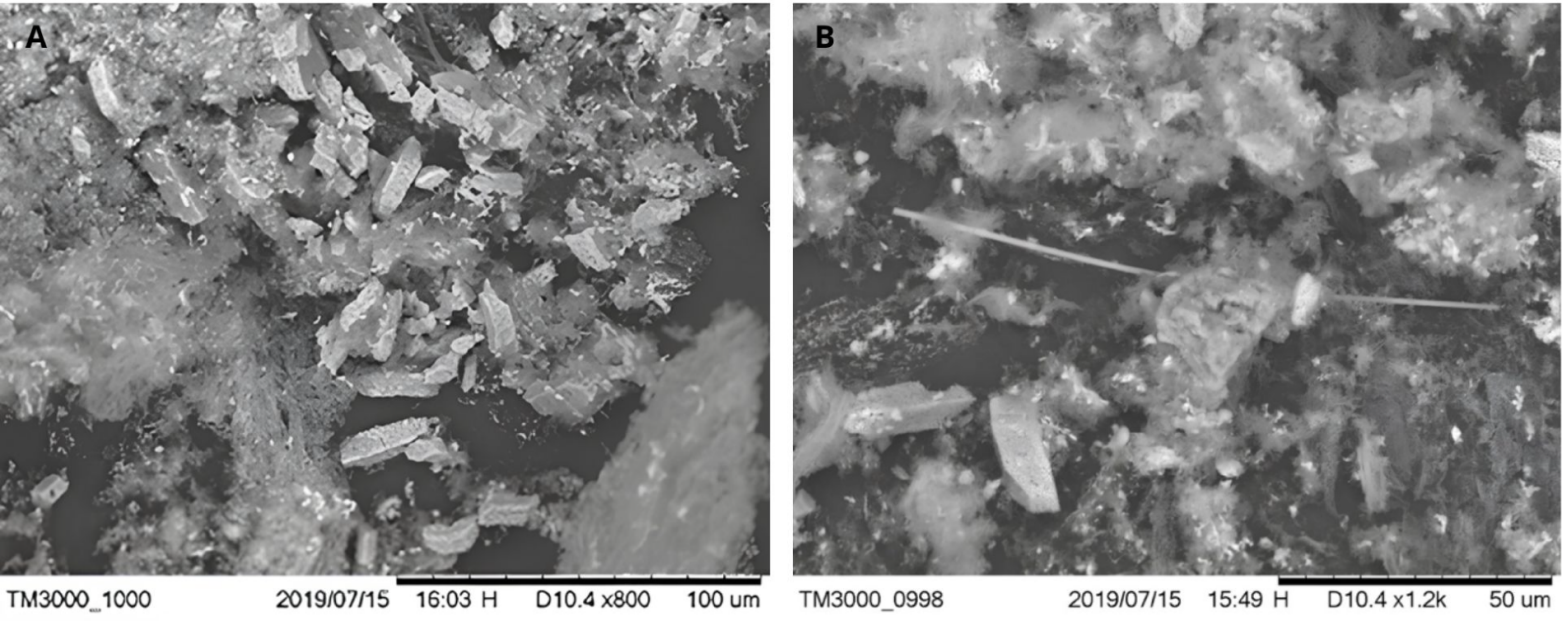
(A) SEM image of bark ashes at 800×. (B) SEM image at 1200× of the region analyzed by EDS.

As shown in Figure 6A, the ash particles present a heterogeneous microstructure, composed of irregular agglomerates, angular fragments, and plate-like or prismatic particles with sharp edges, embedded in a fine particulate matrix. These morphologies suggest the presence of crystalline inorganic phases typically formed during the combustion of lignocellulosic biomass. In Figure 6A, solids with a quadrangular prism morphology are visible, together with lamellar and elongated fragments, likely corresponding to crystals of inorganic compounds such as salts and metal oxides, which typically constitute the main components of ashes derived from lignocellulosic materials.^33^

According to the literature, the primary inorganic compounds in *Eucalyptus* bark ashes include calcium, potassium, sodium, and silicon, with calcium being the most abundant. Calcium is often present as calcium oxalate or calcium carbonate. Specific concentrations of these elements can vary depending on the *Eucalyptus* species and environmental factors.^34,35^

In the higher magnification image (Figure 6B), particles with more defined edges, fractured surfaces, and layered morphologies are observed, although the limited contrast and sharpness of the image restrict a more detailed identification of pore structures or crystallographic features. Nevertheless, the observed morphologies are consistent with mineral-rich ash residues commonly reported for *Eucalyptus* bark combustion products.

The EDS (Figure 7) spectrum reveals detectable peaks for oxygen (O), calcium (Ca), magnesium (Mg), aluminum (Al), potassium (K), silicon (Si), phosphorus (P), sodium (Na), and iron (Fe), which can be beneficial depending on the specific requirements of the soil to which the biochar is applied.^36^

**Figure 7:**
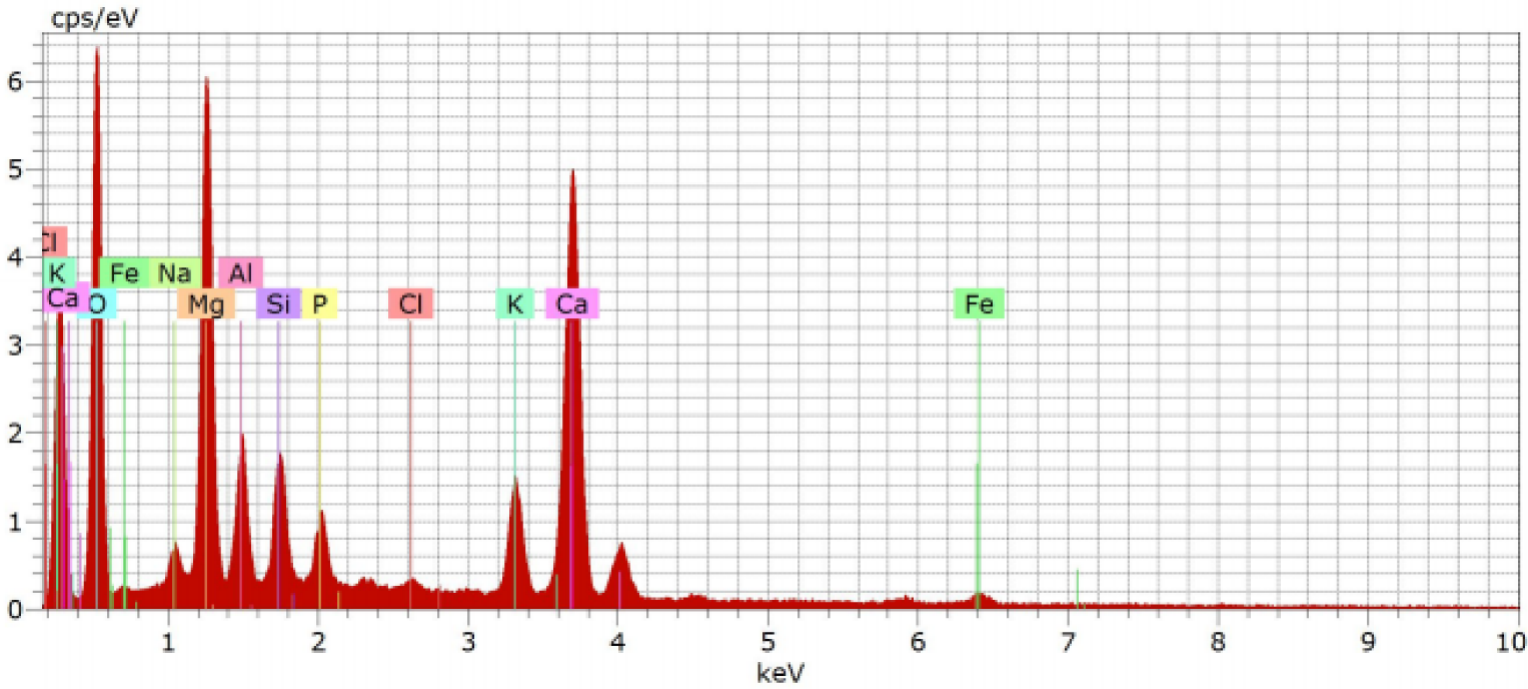
EDS analysis of *Eucalyptus* bark ashes.

### 3.5 XRD

X-ray diffraction (XRD) was employed to assess the crystallinity of the material. The diffractograms of the raw bark and biochar from each treatment are presented in Figure 8.

**Figure 8:**
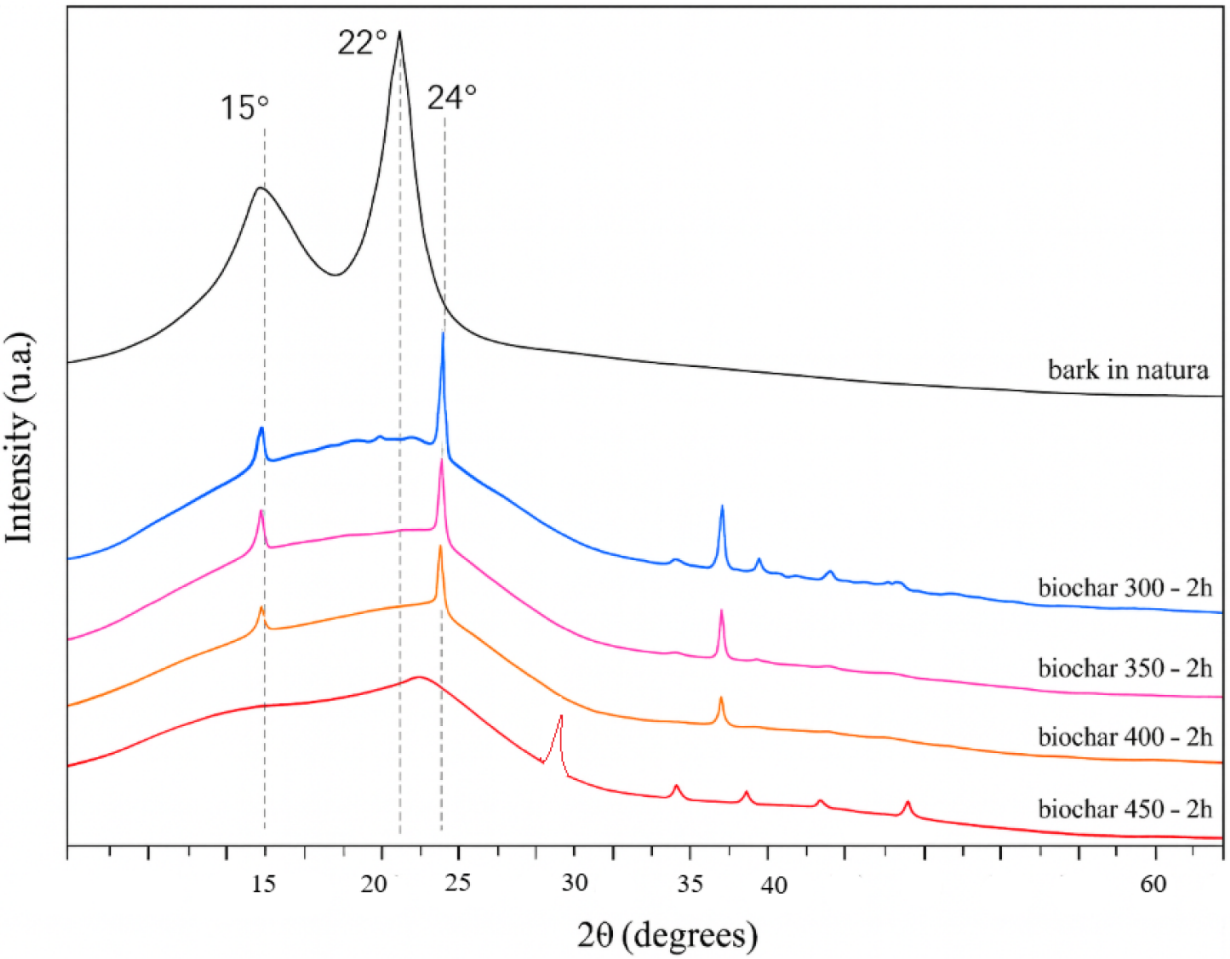
XRD of raw *Eucalyptus* bark and produced biochar.

The diffractogram of the raw *Eucalyptus* bark indicates the presence of a crystalline phase in its composition, due to the presence of cellulose classified as type I (crystalline), identified by peaks at 2θ = 15° and 22°. Other major chemical components of the bark—the amorphous fraction of cellulose (type II), hemicelluloses, and lignin—are predominantly amorphous, as reported in the literature.^37,38^

As the pyrolysis temperature increases, the peaks of type I cellulose lose intensity and acquire the shape of a broad hill in the region of 2θ = 23°, a behavior described by Singh and Raven (2017),^33^ attributed to X-ray scattering when encountering amorphous carbon, or even amorphous silica, in the sample. According to the authors, the presence of aromatic carbon structures also increased as the pyrolysis temperature increased.

The narrow and smaller peaks at 2θ = 15° and 24°, up to the treatment at 400 °C, can be attributed to residual crystalline regions of cellulose that were not completely degraded. According to the literature, cellulose thermal degradation typically begins around 300 °C and reaches its peak near 400 °C.^31,39^

The peaks detected at 15° and 24°, visible in the diffractogram up to the 400 °C biochar, lose definition and become broader and less prominent as the pyrolysis temperature increases, especially in the 450 °C biochar sample, which indicates the loss of crystallinity in the material, characterizing cellulose degradation.^40^

The discrete peaks at 2θ = 28°, 36°, 38° can likely be attributed to inorganic compounds present in the precursor biomass, such as silica, oxides of calcium, magnesium, potassium, and aluminum, among other elements identified by X-ray dispersive spectroscopy (EDS).^41–43^

### 3.6 FTIR

Samples of raw *Eucalyptus* bark and the respective biochar produced in each tested treatment were analyzed by FTIR to assess chemical group changes during pyrolysis. The spectra are shown in Figure 9, and Table 4 summarizes the functional groups associated with each band.

**Table 4:** Identification of Transmittance Bands of Raw *Eucalyptus* Bark and Biochar treatments^30,40,44^

Wavenumber (cm^–1^)	Group	Occurrence
3330	O–H	Lignin and cellulose
2900	–CH_3_, −CH_2_	Carboxylic acids, carbohydrate esters
1730	C=O	Hemicellulose
1508	C=C	Lignin, extractives
1430	C–H	Cellulose
1370	C–H	Hemicellulose
1315	C–H	Cellulose
1200	C–O	Lignin and carbohydrates
1150	C–O–C	Cellulose and hemicellulose
1100	C–O	Lignin and carbohydrates
1025	C–O	Carbohydrates
895	C–H	Amorphous cellulose (C–H deformation)
870	C–H	Aromatic ring/lignin
780	C–H	Aromatic ring/lignin

**Figure 9:**
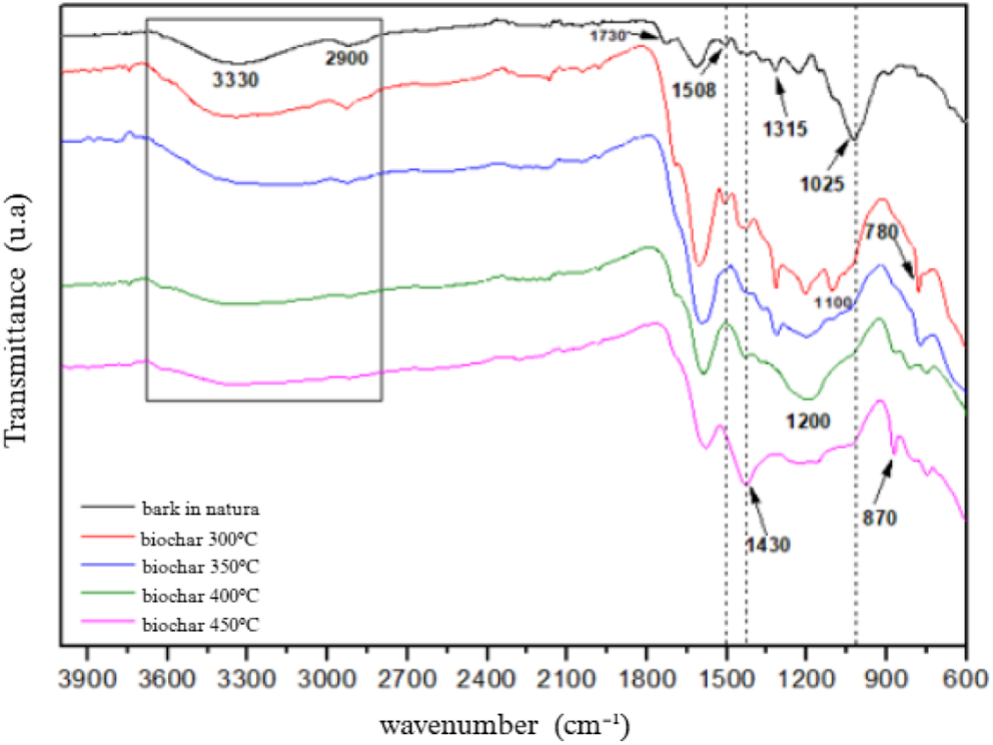
FTIR spectra of raw *Eucalyptus* bark and biochar in the 4000–600 cm^–1^ region.

The raw *Eucalyptus* bark spectrum contains two main regions. The first includes O–H stretching at 3300 cm^–1^, associated with hydroxyl groups in lignin and cellulose, and C–H stretching near 2900 cm^–1^, related to methyl groups in carboxylic acids, esters, and similar compounds. The second region, the “fingerprint” region (1800–800 cm^–1^), includes characteristic vibrations of wood components. The 1510 cm^–1^ band corresponds mainly to C=C in lignin, but can also include contributions from tannin aromatic rings.^44^

Bands around 1430, 1315, 1230, and 1100 cm^–1^ correspond to C–H and C–O groups in carbohydrates, cellulose, and lignin. Bands at 1730, 1370, 1150, and 1023 cm^–1^ arise from C=O, C–H, C–O–C, and C–O vibrations, with 1730 cm^–1^ marking C=O in acetyl and carboxyl groups of hemicelluloses.

The band at 1430 cm^–1^ increases with pyrolysis temperature, indicating aromatic C–H vibrations in crystalline cellulose. The 895 cm^–1^ band marks C–H deformation in amorphous cellulose. A strong band near 875 cm^–1^ appears at 400 °C and additional bands below 800 cm^–1^ emerge from 300 °C onward, associated with aromatic C–H stretching in lignin-derived structures.

Increasing pyrolysis temperature caused broadening and reduction of the O–H band, indicating loss of hydroxyl groups from compounds such as fatty acids, phenols, and alcohols. Bands related to carbohydrates and hemicelluloses (e.g., 2900, 1510, 1370, 1315, 1230 cm^–1^) progressively disappeared in the fingerprint region. Conversely, aromatic lignin bands (C=C and C–H in benzene rings) became more prominent due to their higher thermal resistance.

The degradation of biomass components follows their thermal stability: hemicelluloses degrade around 300 °C, cellulose peaks near 400 °C, and lignin degrades over a wide range (300–900 °C) due to its cross-linked structure.^45,46^

The intensification and emergence of bands from 800 cm^–1^ indicate aromatization of the material during pyrolysis.^47^ The persistence of the 1430 cm^–1^ band in all spectra suggests the presence of residual cellulose even in 450 °C biochar.^48^

### 3.7 TGA

Samples of the bark and the produced biochar were subjected to thermogravimetric analysis (TGA) to evaluate their thermal behavior before and after pyrolysis and to assess the effects of pyrolytic conversion on the material. In Figure 10, the thermogravimetric (TGA) and derivative thermogravimetry (DTG) curves, under inert N_2_ atmosphere, of raw *Eucalyptus* bark and the treatments are shown.

**Figure 10:**
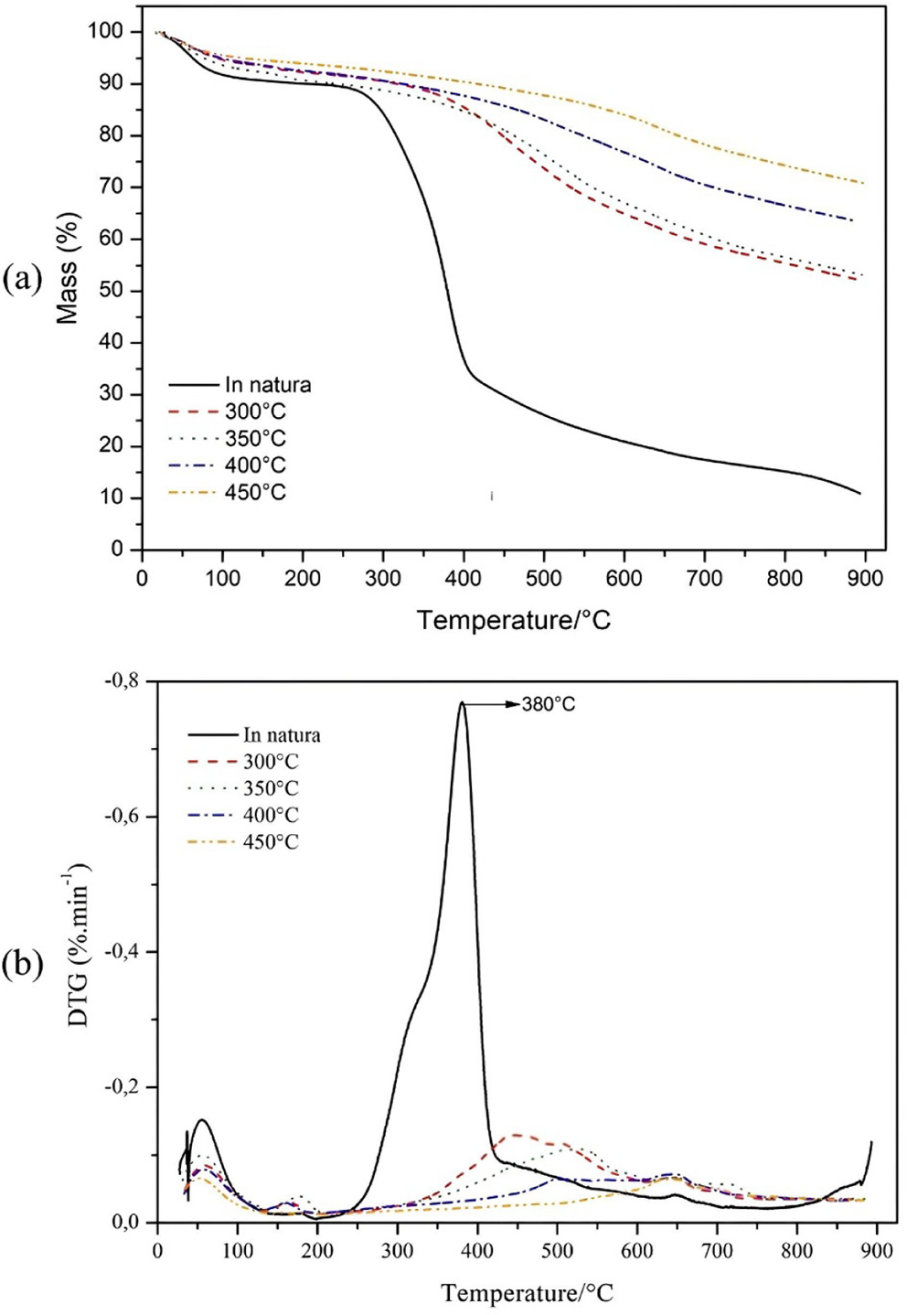
TGA and DTG curves under inert atmosphere (N_2_) and heating rate of 10 °C·min^–1^, of raw *Eucalyptus* bark and treatments with 2 h residence time: (a) TGA curves and (b) DTG curves.

Table 5 presents extrapolated initial and final temperatures, Tonset and Tendset, respectively; the temperature at the maximum degradation rate, Tmáx; corresponding mass loss percentages per event; and the final residual mass percentage.

**Table 5:** TGA Analysis Events, Tonset, Tmáx, Tendset, Mass Loss per Event, and Residual Mass

Sample	Event	Tonset (°C)	Tmáx (°C)	Tendset (°C)	% Mass Loss	% Final Residual Mass
In natura	I	27.3	56.3	87.6	7.8	-
	II	276.4	380	400	55.5	-
	III	>400	-	832.7	25.7	10.9
300 °C	I	29.6	60.1	90.2	5.5	-
	II	381.3	450	614.1	31.4	52.2
350 °C	I	32.5	58.6	86.5	5.9	-
	II	400.6	525	723	27.2	53.0
400 °C	I	40.4	58.7	100.2	5.8	-
	II	444.4	503–640	708.2	17.26	63.6
450 °C	I	31.01	53.5	95.0	4.5	-
	II	553.2	643	714.5	9.2	70.8

For raw *Eucalyptus* bark and all analyzed treatments, the first event corresponds to the release of moisture from the samples. A Tmáx for the bark at 380 °C was identified, which is particularly relevant to guide the most suitable and feasible conditions in the pyrolysis process, in addition to informing the effects of thermal treatment on the material and chemical transformations.^31^

From the second event, the distinction between the thermal decomposition curve of the raw material and the biochar becomes evident. In the curves of biochar samples, Figure 10a, mass loss occurs in a single main event, starting at temperatures near or above 400 °C Consequently, higher pyrolysis temperatures correspond to higher initial degradation temperatures in the TG curves.

The second stage of the analysis for biochar does not exhibit a sharp inflection of mass loss, indicating that the thermal decomposition of these treated materials occurs more gradually, which confers greater thermal stability during the heating process. Similar thermal behavior of biochar compared to its precursor biomass has been reported by Azargohar et al. (2014).^40^

Analyzing the percentages of final residual masses in Table 5, indicates that treatments at higher pyrolysis temperatures conferred greater resistance to thermal degradation. In this sense, samples treated at 300 and 350 °C retained about 50% of the initial total mass at the end of the analysis; treatments at 400 and 450 °C obtained percentages of approximately 60% and 70%, respectively. In contrast, raw *Eucalyptus* bark retained only 10.9% of its mass.

In the DTG curve of raw *Eucalyptus* bark, Figure 10b, there is a shoulder to the right of the main peak, around 315 °C, which can be attributed to hemicellulose degradation and is not observed in biochar curves. Tmáx at 380 °C was consistent with literature values for cellulose, around 400 °C.^31^ Notably, in the DTG curves of the treatments, Tmáx shifts to higher temperatures and peaks broaden.

In the DTG curve of the 400 °C biochar, a pronounced peak appears at 640 °C and another subtle peak around 500 °C, which may indicate residual cellulose; it is not observed in the 450 °C biochar DTG.

The Tmáx at 643 °C for the 450 °C biochar is likely associated with lignin macromolecule degradation, although no exact degradation temperature is established, only a range from 300 to 900 °C.^39^ Figure 11 shows TGA and DTG curves as a function of time (min), resulting from isothermal tests with *Eucalyptus* bark, a technique used to simulate the thermal behavior of the material under the studied pyrolysis conditions.

**Figure 11:**
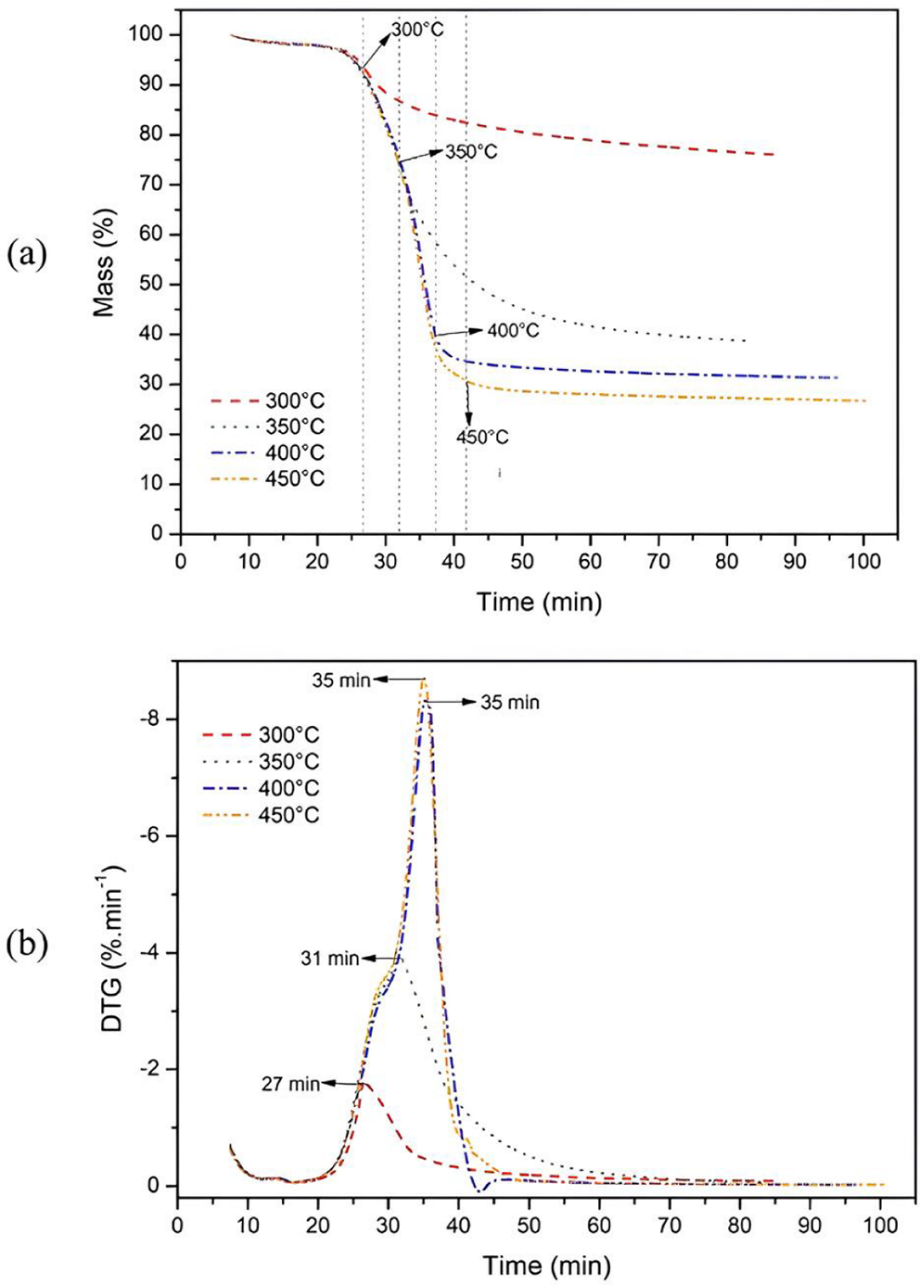
Isothermal TGA (a) and DTG (b) curves of raw *Eucalyptus* bark at 300 °C, 350 °C, 400 and 450 °C for 60 min under inert N_2_ atmosphere and heating rate of 10 °C·min^–1^.

Observing the isothermal curves, Figure 11a,b, it can be inferred that the maximum degradation temperature of the bark was reached at 400 °C, corroborating the Tmáx indicated in the raw *Eucalyptus* bark DTG curve in Figure 10b. From this temperature, a plateau forms, indicating that the mass loss process becomes constant, stabilizing over the subsequent 60 min.

Nonetheless, in the 300 and 350 °C tests, the curves also tend to stabilize, as these these temperatures there is not enough energy to advance the process occurring at 400 °C, such as marked cellulose thermal degradation. On the other hand, in the 450 °C test, there was a 4.6% higher sample mass loss compared to the 400 °C test, and probably greater mass losses would be observed as the pyrolysis temperature increases.

The difference in gravimetric yield, i.e., the mass percentage of material converted into biochar, between the 300 and 350 °C tests is notable. In the first case, 76% biochar was produced, while in the second, 38.7%, a difference attributable to the pyrolysis conditions. At 300 °C, the fraction corresponding to hemicelluloses degrades, whereas at 350 °C, cellulose degradation begins, progressing to higher temperatures, as seen in the other treatments analyzed.

By correlating the FTIR and TGA results, it was observed that the formation of aromatic structures became evident above 300 °C, when bands associated with aromatic compounds appeared, confirming material carbonization. TGA curves reinforced this trend, showing that treatments presented peaks at higher temperatures, indicating greater thermal stability due to the presence of lignin aromatic bonds.

Indeed, the diffractograms allowed the prediction, of the thermal thermal behavior and structural changes of the pyrolyzed bark, as the cellulose crystallinity peaks disappeared in the B450 sample, confirming that cellulose underwent thermal conversion around at 400 °C.

### 3.8 Integrated Interpretation of XRD, FTIR, and TGA

To clarify the consistency among the XRD, FTIR, and TGA results, it is important to highlight how the thermal degradation stages of the main biomass constituents reflect across the three techniques. Hemicelluloses degrade mainly between 250–300 °C, which corresponds to the disappearance of carbohydrate-related FTIR bands (e.g., 1730, 1370, and 1230 cm^–1^) and to the shoulder observed near 315 °C in the DTG curve of the raw bark.

Cellulose degradation occurs predominantly between 300–400 °C, in agreement with the progressive reduction of the crystalline cellulose peaks at 2θ ≈ 15° and 22° in the XRD patterns and the attenuation of FTIR bands at 1430, 1315, and 1100 cm^–1^.

Lignin, in turn, shows a much broader degradation range (300–900 °C), reflected in the persistence and intensification of aromatic FTIR bands (e.g., 1510 and 875–780 cm^–1^) and in the high-temperature DTG peaks (500–650 °C) observed for biochars. The disappearance of cellulose crystallinity in the 450 °C biochar, together with the emergence of aromatic structures in FTIR and the shift of TGA degradation events to higher temperatures, confirms the coherent evolution of structural and chemical changes throughout pyrolysis.

## 4 Conclusions

The production of biochar from *Eucalyptus* bark under four treatments (300, 350, 400, and 450 °C) with a residence time of 2 h was successful, achieving the objectives of production and physicochemical characterization. All treatments yielded gravimetric yields above 30% and an average fixed carbon content greater than 60%, demonstrating the effectiveness of the adopted residence time. Pyrolysis enhanced carbon concentration in the biochar, resulting in increased thermal stability when compared to raw *Eucalyptus* bark. XRD and FTIR analyses revealed chemical modifications in the biochar with increasing pyrolysis temperature, including residual cellulose and the development of aromatic structures attributed to lignin. Thermogravimetric analysis confirmed that the biochar exhibits greater thermal resistance and stability than the original biomass. Among the treatments, the biochar produced at 450 °C stood out for its high fixed carbon content, elevated pH, reduced volatile matter, and enhanced thermal stability, indicating this condition as the most promising among those evaluated. These properties are particularly relevant for soil application, as the higher pH and greater thermal stability contribute to long-term carbon persistence, potential liming effects, and improvements in soil structure. Future studies should include soil-based experiments to evaluate parameters such as pH adjustment capacity, cation exchange capacity, water retention, and effects on seed germination and early plant growth, which would help validate the suitability of the 450 °C biochar for agricultural applications.
